# Elevated CO_2_ Shifts Photosynthetic Constraint from Stomatal to Biochemical Limitations During Induction in *Populus tomentosa* and *Eucalyptus robusta*

**DOI:** 10.3390/plants14010047

**Published:** 2024-12-27

**Authors:** Xianhui Tang, Jie Zhao, Jiayu Zhou, Qingchen Zhu, Xiyang Sheng, Chao Yue

**Affiliations:** 1 The Research Center of Soil and Water Conservation and Ecological Environment, Chinese Academy of Sciences and Ministry of Education, Yangling 712100, China; tangxhucas@163.com; 2 Institute of Soil and Water Conservation, Chinese Academy of Sciences and Ministry of Water Resources, Yangling 712100, China; 3 University of Chinese Academy of Sciences, Beijing 100049, China; 4 Shandong Provincial Key Laboratory of Water and Soil Conservation and Environmental Protection, College of Resources and Environment, Linyi University, Linyi 276000, China; haiyanpeihongshu@163.com (J.Z.); gilgameshdisney@gmail.com (Q.Z.); 13165151237@163.com (X.S.); 5 State Key Laboratory of Soil Erosion and Dryland Farming on the Loess Plateau, Northwest A&F University, Yangling 712100, China

**Keywords:** carbon dioxide, photosynthetic induction, limitation analysis, photochemistry, non-photochemical quenching, stomatal conductance

## Abstract

The relative impacts of biochemical and stomatal limitations on photosynthesis during photosynthetic induction have been well studied for diverse plants under ambient CO_2_ concentration (*C*_a_). However, a knowledge gap remains regarding how the various photosynthetic components limit duction efficiency under elevated CO_2_. In this study, we experimentally investigated the influence of elevated CO_2_ (from 400 to 800 μmol mol^–1^) on photosynthetic induction dynamics and its associated limitation components in two broadleaved tree species, *Populus tomentosa* and *Eucalyptus robusta*. The results show that elevated CO_2_ increased the steady-state photosynthesis rate (*A*) and decreased stomatal conductance (*g*_s_) and the maximum carboxylation rate (*V*_cmax_) in both species. While *E. robusta* exhibited a decrease in the linear electron transport rate (*J*) and the fraction of open reaction centers in photosynthesis II (*q*_L_), *P. tomentosa* showed a significant increase in non-photochemical quenching (NPQ). With respect to non-steady-state photosynthesis, elevated CO_2_ significantly reduced the induction time of *A* following a shift from low to high light intensity in both species. Time-integrated limitation analysis during induction revealed that elevated CO_2_ reduces the relative impacts of stomatal limitations in both species, consequently shifting the predominant limitation on induction efficiency from stomatal to biochemical components. Additionally, species-specific changes in *q*_L_ and NPQ suggest that elevated CO_2_ may increase biochemical limitation by affecting energy allocation between carbon fixation and photoprotection. These findings suggest that, in a future CO_2_-rich atmosphere, plants productivity under fluctuating light may be primarily constrained by photochemical and non-photochemical quenching.

## 1. Introduction

CO_2_ and light are essential substrates for photosynthesis that drive plant growth and productivity by converting light energy into chemical energy. While the effects of fluctuations in CO_2_ concentration and light levels on photosynthesis have been extensively studied under steady-state conditions [1,2,3,4], natural environments rarely maintain such conditions. Instead, they exhibit rapid fluctuations in environmental drivers, especially irradiance [5,6,7]. The rate of photosynthesis (*A*) changes following a shift from low to high light, a process termed “photosynthetic induction”, which has garnered increasing attention as it more closely reflects the kinetics of photosynthesis under natural conditions [7,8,9,10]. Studies have shown that photosynthetic induction is primarily governed by three physiological processes: the rate of light-activated electron transport, the activation of Rubisco in response to light levels and CO_2_ concentration, and the supply of CO_2_ through stomatal movement [10,11,12]. As these processes are sensitive to CO_2_ levels, gaps remain in understanding how changes in the activation of carboxylation enzymes and stomatal movement influence photosynthetic induction under elevated CO_2_.

In the field, particularly within complex ecosystems such as forests, leaves experience large spatial and temporal fluctuations in irradiance levels and CO_2_ concentrations [13,14,15]. Factors such as canopy structure, cloud cover, solar angle, and wind-induced leave movement contribute to these variations [5,9]. Under these conditions, leaves must balance energy supply and demand to maximize carbon assimilation while protecting the photosynthesis apparatus from potential damage [16,17,18,19]. This balance is primarily achieved through adjustment in photochemical quenching (PQ) and non-photochemical quenching (NPQ), which manage light energy absorption and use within chloroplasts [12,20]. PQ involves chlorophyll-driven electron transport that produces ATP and NADPH, reflecting the dynamics of the open fraction of photosynthesis II (PSII) reaction centers and the reduction–oxidation state of the primary quinone acceptor of PSII (*Q*_A_) [18,21]. The fraction of open PSII reaction centers, denoted as *q*_L_ in the lake model, assumes fully connected photosynthetic units sharing energy freely [22]. NPQ, a protective mechanism, dissipates excess absorbed light energy as heat, safeguarding the photosynthetic machinery from damage and competing with photochemical quenching [17]. During photosynthetic induction, the slow activation of stomatal movement and Rubisco limits the diffusion of CO_2_ and carboxylation rate, resulting in a delayed light reaction response and potentially reducing photosynthetic productivity [23,24]. Dynamic changes in *q*_L_ and NPQ are crucial for plants adapting to varying light conditions as they influence photosynthetic productivity.

Rising CO_2_ concentrations are predicted to generally enhance photosynthesis while reducing stomatal conductance to water vapor (*g*_s_) under steady-state conditions [4,25,26]. Additionally, the biochemical limitations of photosynthesis shift from Rubisco activity to the rate of RuBP-regeneration [27,28]. During photosynthetic induction, the activation of RuBP-regeneration takes approximately 60 s, whereas full activation of Rubisco requires 8–10 min, and stomatal opening takes even longer [29,30,31,32]. Consequently, slow stomatal opening may limit photosynthesis during induction, while elevated CO_2_ could reduce biochemical limitations, enhancing photosynthetic productivity [33,34,35]. However, the effect of elevated CO_2_ on photosynthetic induction rates remains unclear, with some studies reporting faster induction and others finding no differences [7]. Elevated CO_2_ has also been associated with rapid stomatal opening but a lower maximum *g*_s_ at higher irradiance [33], potentially reducing stomatal limitation. Nonetheless, the relative contributions of biochemical and stomatal limitations on photosynthetic induction under elevated CO_2_ have not been thoroughly analyzed. Furthermore, previous analyses of relative limitations have often assumed Rubisco-limited conditions [10], yet photosynthesis under elevated CO_2_ conditions may be limited by electron transport rate [28]. Since *q*_L_ and NPQ are critical in determining electron transport rate [20], understanding their variation and role in photosynthetic induction under elevated CO_2_ is crucial for elucidating shifts in photosynthetic limitations.

In this study, we aimed to address these gaps by investigating the effects of elevated CO_2_ concentrations (from 400 to 800 μmol mol^–1^) on leaf gas exchange and chlorophyll fluorescence during photosynthetic induction. By conducting these measurements and analyzing relative photosynthetic limitations, we sought to clarify how elevated CO_2_ influences the physiological processes governing plant responses to increasing light levels and quantify the changes in both biochemical limitations and stomatal limitations on photosynthetic induction under elevated CO_2_. We hypothesized that elevated CO_2_ would lead to faster stomatal opening and more efficient activation of photosynthetic processes, thereby reducing the relative limitation imposed by stomatal factors. Additionally, we anticipated that the dynamics of *q*_L_ and NPQ would reflect shifts in energy dissipation pathways and modulated electron transport rate, influencing biochemical limitations. We believe that our findings will provide insights into how plant photosynthesis responds to fluctuating environmental conditions under the future atmospheric CO_2_ scenarios, with implications for ecosystem resilience and agricultural productivity.

## 2. Materials and Methods

### 2.1. Plant Materials and Growth Conditions

Two three-year-old saplings of two species (a deciduous species *Populus tomentosa* Carr. and an evergreen species *Eucalyptus robusta* smith) were chosen for the experiments. These two species are dominant tree species in the evergreen broad-leaved forests in southern China and deciduous forests in northern China, respectively. Saplings with similar heights and basal diameters were grown outdoors in the Campus of Shenzhen University, Shenzhen, China (113°55′ E, 22°31′ N) in pots (25 cm diameter × 32 cm height) filled with a mixture a soil and organic substrate. The plants were irrigated daily and fertilized twice a week with half-strength Hoagland’s solution. After 2 months growth outdoors, plants were moved into laboratory for measurements with room temperature set to 26 °C.

### 2.2. Gas Exchange and Chlorophyll Fluorescence Induction

Gas exchange and chlorophyll fluorescence were measured on at least four replicate individuals per species using a LI-6800 photosynthesis system equipped with leaf multiphase flash chamber fluorometer chamber (6800-01A) (LI-COR, Inc., Lincoln, NE, USA). All measurements were performed on the youngest fully expanded leaves of the main stem. During measurements, the leaf chamber conditions were maintained at a flow rate of 500 μmol s^–1^, relative humidity of 60%, air temperature of 25 °C, and a leaf vapor pressure deficit of 1.2 kPa. Plants were kept in the dark overnight for dark adaptation before measurements. At the start of each measurement, the leaf was placed in the leaf chamber to measure the minimum fluorescence (*F*_o_) and maximum fluorescence (*F*_m_) using rectangular flash, and the dark respiration (*R*_n_) could also be obtained at the same time.

Subsequently, leaves were acclimated to a low photosynthetic photon flux density (PPFD) of 100 μmol m^–2^ s^–1^ (blue: red light ratio set at 10%: 90%) for *c*. 30 min to allow for the achievement of steady-state photosynthesis rate (*A*) and stomatal conductance to water (*g*_s_). Then, a step increasing to the high PPFD level of 1200 μmol m^–2^ s^–1^ for at least 45 min until *A* and *g*_s_ stabilized at a new steady state. During the measurements, gas exchange parameters, steady-state fluorescence (*F*_s_), and maximum fluorescence during a multiphase light-saturating pulse flash ($F_{m}^{\prime }$) were recorded every minute. To assess the effect of elevated CO_2_, CO_2_ levels in the chamber were adjusted to 400 and 800 μmol mol^–1^ with a CO_2_ mixture, representing ambient atmospheric CO_2_ (ambient *C*_a_) and elevated CO_2_ (elevated *C*_a_), respectively.

*F*_s_ and $F_{m}^{\prime }$ can be used to determine actual photochemical efficiency of PSII (*Φ*_PSII_) as follows:

(1) $$\Phi_{\mathrm{PS}\text{II}}=\frac{F_{m}^{\prime }-F_{s}}{F_{m}^{\prime }}$$

Then, the linear electron transport rate (*J*) can be derived as follows:

(2) $$J\;=\Phi_{\mathrm{PSII}}\cdot \mathrm{PPFD}\cdot \alpha \beta$$

where *α* is the leaf absorbance, *β* is the fraction of quanta absorbed by PSII, and the *a*·*β* was determined through a linear correlation between *Φ*_PSII_·PPFD/4 and net photosynthetic rate (*A*), flowing the standard steady-state light response curve under 2% O_2_ and CO_2_ at 1000 μmol mol^–1^ as per Yin et al. (2009) [36].

The fraction of open reaction centers in PSII (*q*_L_) is then calculated as follows [22]:

(3) $$q_{L}=\frac{F_{o}^{\prime }}{F_{s}}\cdot \frac{F_{m}^{\prime }-F_{s}}{F_{m}^{\prime }-F_{o}^{\prime }}$$

where $F_{o}^{\prime }$ is minimum fluorescence under light that can be determined as follows [18]:

(4) $$F_{o}^{\prime }=\frac{F_{o}}{\frac{F_{o}\;-\;F_{m}}{F_{m}}+\frac{F_{o}}{F_{m}^{\prime }}}$$

Non-photochemical quenching (NPQ) is estimated as follows:

(5) $$\mathrm{NPQ}=\frac{F_{m}-F_{m}^{\prime }}{F_{m}^{\prime }}$$

### 2.3. Analysis of Biochemical and Stomatal Limitations to A During Induction

A differential method was applied to quantify the relative limitations of biochemical effects (d*A*_biochem_) and stomatal effects (d*A*_stom_) on the photosynthesis induction, based on Deans et al. (2019 a, b) [10,30]. This method calculates the linearized difference between the final *A* and current state *A* (d*A*_calc_) as the sum of d*A*_biochem_ and d*A*_stom_, under the assumption that leaves would attain the potential *A* if the biochemical activation and stomatal opening were to reach their final states instantaneously:

(6) $${dA}_{\mathrm{calc}}={dA}_{\mathrm{biochem}}+{dA}_{\mathrm{stom}}$$

If we further assume that biochemical limitation mainly relates to the Rubisco limitation rather than electron transport rate limitation, d*A*_biochem_ and d*A*_stom_ can be expressed as follows:

(7) $$dA_{\mathrm{biochem}}=\frac{\text{∂}A}{\text{∂}V_{cmax}}dV_{cmax}$$

(8) $$dA_{\mathrm{stom}}=\frac{\text{∂}A}{\text{∂}g_{sc}}dg_{sc}$$

where *V*_cmax_ is the maximum carboxylation rate, *g*_sc_ is the stomatal conductance to CO_2_ with a convert factor of 1.6 to *g*_s_ (i.e., *g*_s_ = 1.6 *g*_sc_), and d*V*_cmax_ and d*g*_sc_ represent the differences between the final and current values of *V*_cmax_ and *g*_sc_. According to the biochemical photosynthesis model of Farquhar et al. (1980) [1], under Rubisco-limited condition, neglecting the resistance of mesophyll, the assimilate rate is

(9) $$A\;=V_{cmax}\frac{C_{i}-\Gamma^{*}}{C_{i}+K_{M}}-R_{d}$$

where *C*_i_ is the intercellular CO_2_ concentration, *K*_M_ is the Michaelis–Menten coefficient for Rubisco at a 21% O_2_, Γ^*^ is the CO_2_ compensation point in the absence of mitochondrial respiration, and *R*_d_ is day respiration, assumed to be half of the dark respiration [37] (i.e., *R*_d_ = *R*_n_/2). Variation in *V*_cmax_ during the photosynthetic induction can be calculated from rearranging Equation (9) as follows:

(10) $$V_{cmax}=\frac{\left(A+R_{d}\right)\left(C_{i}+K_{M}\right)}{\left(C_{i}-\Gamma^{*}\right)}$$

To obtain the partial derivatives of Equations (7) and (8), we need to express *A* as a function of *V*_cmax_ and *g*_sc_, and then derive its implicit differentiation. By combining Equation (9) with Fick’s Law [38]:

(11) $$A=g_{sc}\left(C_{a}-C_{i}\right)$$

We obtain a quadratic equation regarding *A*:

(12) $$\frac{1}{g_{sc}}A^{2}-\left[\left(V_{cmax}-R_{d}\right)\left(\frac{1}{g_{sc}}\right)+\left(C_{a}+K_{M}\right)\right]A\;+V_{cmax}\left(C_{a}-\Gamma^{*}\right)-R_{d}\left(C_{a}+K_{M}\right)=0$$

The partial derivatives of Equations (7) and (8) are given by differentiating Equation (12) with *V*_cmax_ and *g*_s_, respectively, as follows:

(13a) $$\frac{\text{∂}A}{\text{∂}V_{cmax}}=\frac{C_{a}-\Gamma^{*}-\left(\frac{1}{g_{s}}\right)A}{\left(V_{cmax}-R_{d}\right)\left(\frac{1}{g_{\mathrm{sc}}}\right)+C_{a}+K_{M}-\;2\left(\frac{1}{g_{\mathrm{sc}}}\right)A}$$

(13b) $$\frac{\text{∂}A}{\text{∂}g_{\mathrm{sc}}}=\frac{\frac{A}{g_{\mathrm{sc}}^{2}}(V_{cmax}-R_{d}-A)}{\left(V_{cmax}-R_{d}\right)\left(\frac{1}{g_{\mathrm{sc}}}\right)+C_{a}+K_{M}-\;2\left(\frac{1}{g_{\mathrm{sc}}}\right)A}$$

Then, the time-integrated total forgone assimilation of can be calculated by [31]

(14) $$\Delta C_{\mathrm{stom}/\mathrm{biochem}/\mathrm{calc}}=\int_{0}^{t_{f}}dA_{\mathrm{stom}/\mathrm{biochem}/\mathrm{calc}}dt$$

where *t*_f_ refers to the time of steady-state measurement. The relative limitations imposed by *g*_s_ and *V*_cmax_ through the photosynthetic induction can be determined by

(15) $$\sigma_{\mathrm{stom}/\mathrm{biochem}}=\frac{\Delta C_{\mathrm{stom}/\mathrm{biochem}}}{\Delta C_{\mathrm{calc}}}$$

However, under high CO_2_ concentration, photosynthetic limitation may shift from Rubisco-limited state to electron transport rate-limited state [28], i.e., the above equations used to calculate biochemical limitation may not be applicable for elevated CO_2_ conditions. Hence, according to the biochemical photosynthesis model of Farquhar et al. (1980) [1], electron transport rate-limited photosynthesis rate (*A*_j_) is

(16) $$A\;=J\frac{C_{i}-\Gamma^{*}}{{4C}_{i}+{8\Gamma }^{*}}-R_{d}$$

Consequently, d*A*_biochem_ can be determined as follows:

(17) $$dA_{\mathrm{bio}\mathrm{chem}}=\frac{\text{∂}A}{\text{∂}J}dJ$$

Then, by combining Equation (16) with Equation (11), a quadratic equation regarding *A* can be written:

(18) $$\left(\frac{4}{g_{sc}}\right)A^{2}-\left[\left(J-4R_{d}\right)\left(\frac{1}{g_{\mathrm{sc}}}\right)+4\left(C_{a}+2\Gamma^{*}\right)\right]A-J\left(C_{a}-\Gamma^{*}\right)+4R_{d}\left(C_{a}+2\Gamma^{*}\right)=0$$

The partial derivative of Equation (17) is given by differentiating Equation (18) with *J*, as follows:

(19) $$\frac{\text{∂}A}{\text{∂}J}=\frac{C_{a}-\Gamma^{*}-\left(\frac{1}{g_{\mathrm{sc}}}\right)A}{\left(J-4R_{d}\right)\left(\frac{1}{g_{\mathrm{sc}}}\right)+{4(C}_{a}+2\Gamma^{*})-\;8\left(\frac{1}{g_{\mathrm{sc}}}\right)A}$$

Under elevated CO_2_ conditions, Equation (19) is applied to calculate the relative limitations imposed by biochemical effects.

The photosynthetic induction response curve, which is used to calculate the induction kinetics for a given photosynthetic parameter, follows an exponential model as described by Mott and Woodrow (2000) [29]:

(20) Ft=Fi+Fi−Ffe−tτ

where *F*(*t*), *F*_i_, *F*_f_ represent the values of photosynthetic parameters (*A*, *g*_s_, *V*_cmax_, *J*) at the current time (*t*), the initial state, and the final steady state, respectively. *τ* refers to the time constant for the increase in *F*(*t*), mathematically representing the time at which *F*(*t*) reaches 63% of its final steady-state condition [39] ($\mathrm{when}\;\frac{F\left(t\right)-F_{i}}{F_{f}-F_{i}}=1-e^{-1}\approx 0.63$). Consequently, the time required to reach 90% of the steady-state value, denoted as *t*_90_, is calculated as follows:

(21) $$t_{90}=-\tau \log_{e}\left(0.1\right)$$

### 2.4. Statistical Analysis

For a given parameter, normality of distributions was performed through Shapiro–Wilk tests. Because the data were paired and did not meet the assumption of normality for parametric tests, we chose the Wilcoxon signed-rank test to compare the parameters between the ambient and elevated C_a_. A significance level of 0.05 was used for all statistical tests. All statistical analyses were conducted using R language and environment (R Core Team 2023) [40].

## 3. Results

### 3.1. Temporal Responses of Parameters from Leaf Gas Exchange and Chlorophyll Fluorescence

**Figure 1:**
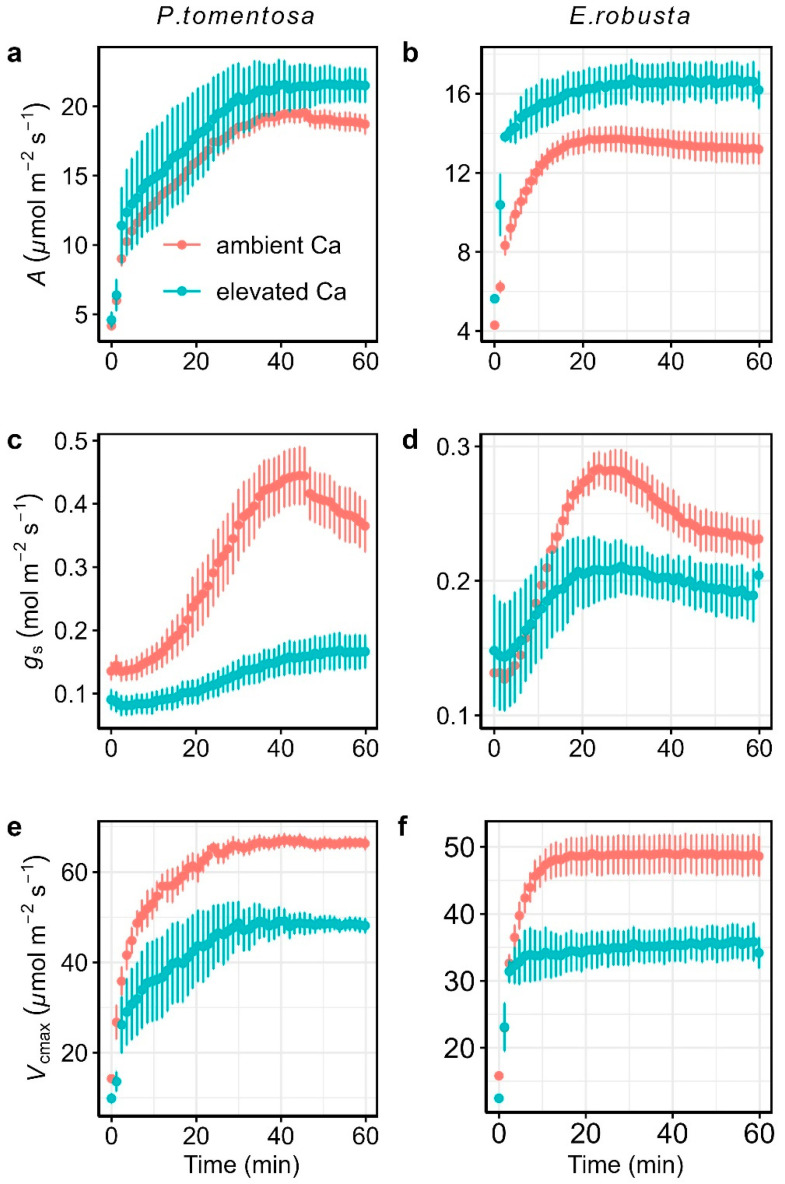
Temporal response of leaf gas exchange parameters to photosynthetic induction under ambient CO_2_ (ambient *C*_a_, red) and elevated CO_2_ (elevated *C*_a_, cyan) conditions for *Populus tomentosa* (**left panel**) and *Eucalyptus robusta* (**right panel**). Parameters include net photosynthesis rate (*A*; (**a**,**b**)), stomatal conductance to water vapor (*g*_s_; (**c**,**d**)), and maximum carboxylation rate (*V*_cmax_; (**e**,**f**)). Vertical bars in each plot represent the standard error from 4 replicate leaves.

**Figure 2:**
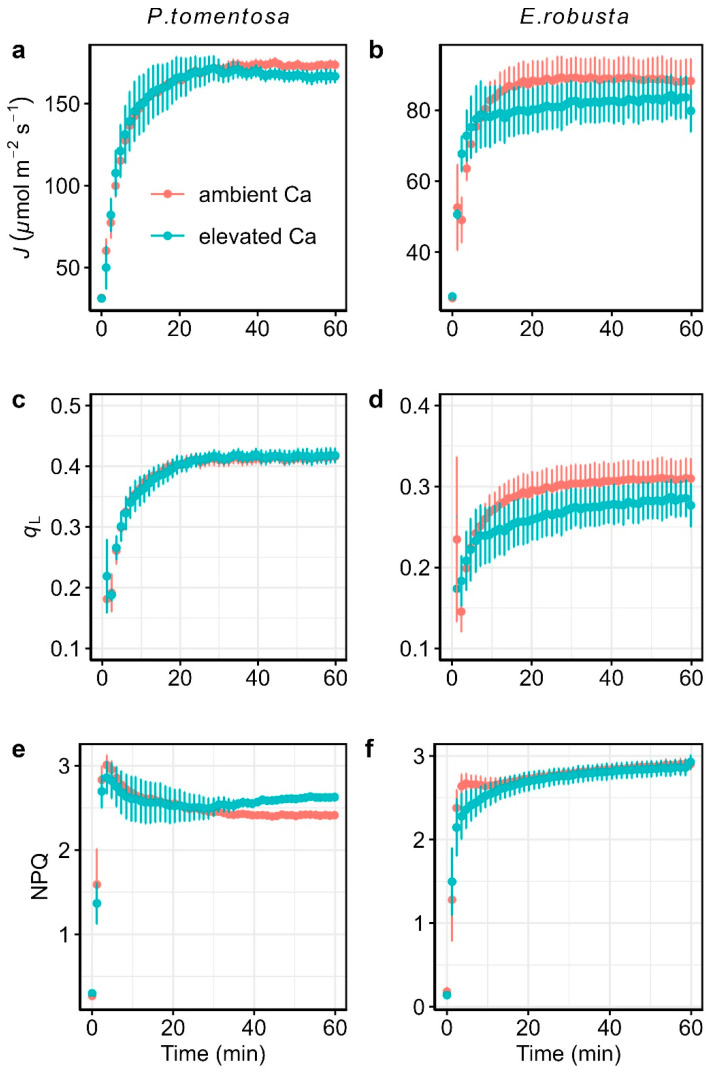
Temporal response of chlorophyll fluorescence-derived parameters to photosynthetic induction under ambient CO_2_ (ambient *C*_a_, red) and elevated CO_2_ (elevated *C*_a_, cyan) conditions for *Populus tomentosa* (**left panel**) and *Eucalyptus robusta* (**right panel**). Parameters include linear electron transport rate (*J*; (**a**,**b**)), the fraction of open reaction centers in PSII (*q*_L_; (**c**,**d**)), and non-photochemical quenching (NPQ; (**e**,**f**)). Vertical bars in each plot represent the standard error from 4 replicate leaves.

To evaluate the effects of elevated CO_2_ on photosynthetic induction, we monitored the temporal responses of leaf gas exchange parameters (Figure 1) and chlorophyll fluorescence-derived parameters (Figure 2) after dark adaptation in *Populus. tomentosa* and *Eucalyptus. robusta*. The leaf gas exchange parameters measured included net photosynthesis rate (*A*), stomatal conductance to water vapor (*g*_s_), and the maximum rate of Rubisco activity (*V*_cmax_). Chlorophyll fluorescence-derived parameters included the linear electron transport rate (*J*), the fraction of open reaction centers in PSII (*q*_L_), and non-photochemical quenching (NPQ). As shown in Figure 1, after transitioning from low to high light, *A*, *g*_s_, and *V*_cmax_ gradually increase over time before reaching steady-state levels. Unlike *A* and *V*_cmax_, which reached and maintained peak values, *g*_s_ initially increased to the maximum before declining, resulting in final measurements lower than the peak value in both *P. tomentosa* and *E. robusta*. Under elevated *C*_a_, steady-state *A* increased (average increases of 11.66% increasing in *P. tomentosa* and 27.99% in *E. robusta*), while steady-state *g*_s_ and *V*_cmax_ declined (mean declines of 58.54% and 21.74% in *g*_s_ and 27.99% and 26.56% in *V*_cmax_ for *P. tomentosa* and *E. robusta*, respectively) (Table S1). For chlorophyll fluorescence-derived parameters, as shown in Figure 2, *J* and *q*_L_ gradually increased with time, whereas NPQ rapidly peaked before gradually declining to steady-state values. Compared to ambient *C*_a_, there were no significant differences in the steady-state values of *J* and *q*_L_ under elevated *C*_a_ in both species. However, *P. tomentosa* showed significantly higher NPQ under elevated C_a_.

**Figure 3:**
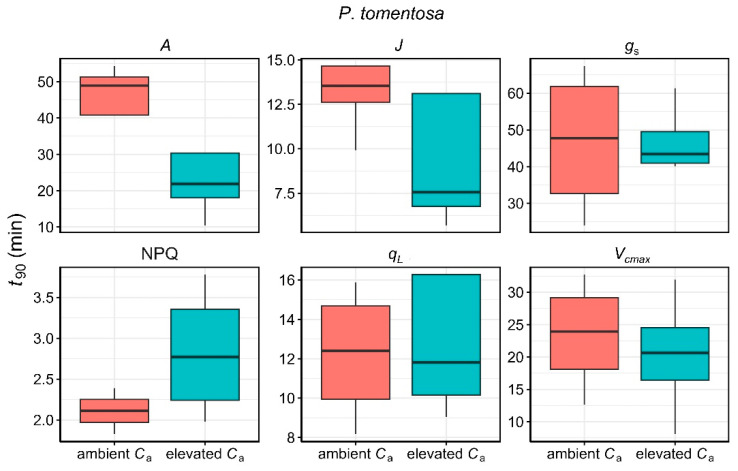
Comparison of the time to reach 90% of the steady-state values (*t*_90_) for *A*, *J*, *g*_s_, NPQ, *q*_L_, and *V*_cmax_ in *Populus tomentosa* under ambient (red) and elevated *C*_a_ (cyan) conditions.

**Figure 4:**
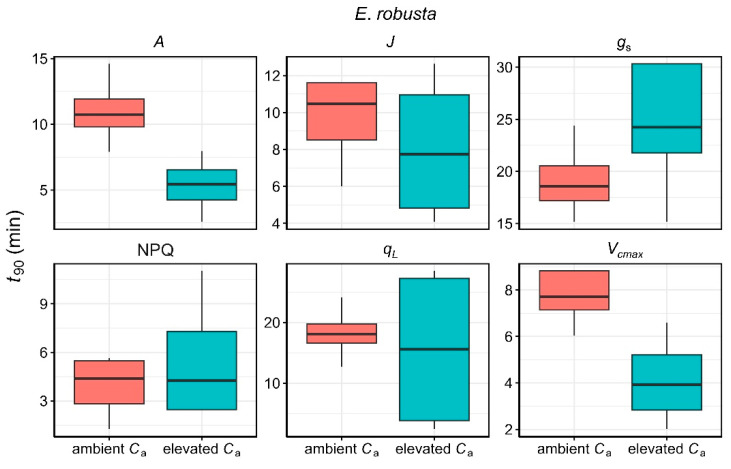
Comparison of the time to reach 90% of the steady-state values (*t*_90_) for *A*, *J*, *g*_s_, NPQ, *q*_L_, and *V*_cmax_ in *E. robusta* under ambient (red) and elevated *C*_a_ (cyan) conditions.

Regarding the time required to reach 90% of the steady-state value (*t*_90_), there were no statistically significant differences between ambient and elevated *C*_a_ for either species (Figure 3 and Figure 4). Nonetheless, elevated *C*_a_ conditions resulted in noticeably faster responses in *A*, *V_cmax_*, and *J* in both *P. tomentosa* and *E. robusta*, with substantial variability observed in the response of *g*_s_, *q*_L_, and NPQ.

### 3.2. Photosynthetic Limitations Analysis

**Figure 5:**
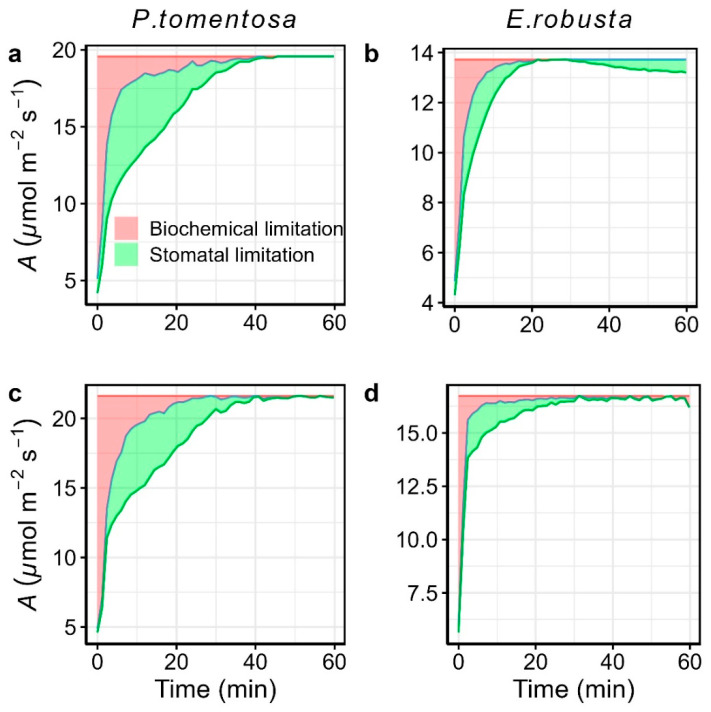
Biochemical and stomatal limitations to photosynthesis during photosynthetic induction under ambient *C*_a_ (**upper panel**; (**a,b**)) and elevated *C*_a_ (**lower panel**; (**c,d**)) for *Populus tomentosa* (**left panel**; (**a,c**)) and *Eucalyptus robusta* (**right panel**; (**b,d**)). At each time point, the deviation of *A* from the final steady-state condition (d*A*_calc_) is divided into biochemical limitation (d*A*_biochem_, red area) and stomatal limitation (d*A*_stom_, green area). d*A*_calc_ represents the linearized difference between steady-state *A* and the current state *A* (d*A*); hence, d*A*_biochem_ and d*A*_stom_ in the figures are normalized by multiplying d*A*/d*A*_calc_ for visual comparison. Under ambient *C*_a_, d*A*_biochem_ is calculated based on the derivations of Rubisco-limited photosynthesis, while under elevated *C*_a_, it is calculated based on the derivations of electron transport-limited photosynthesis.

**Figure 6:**
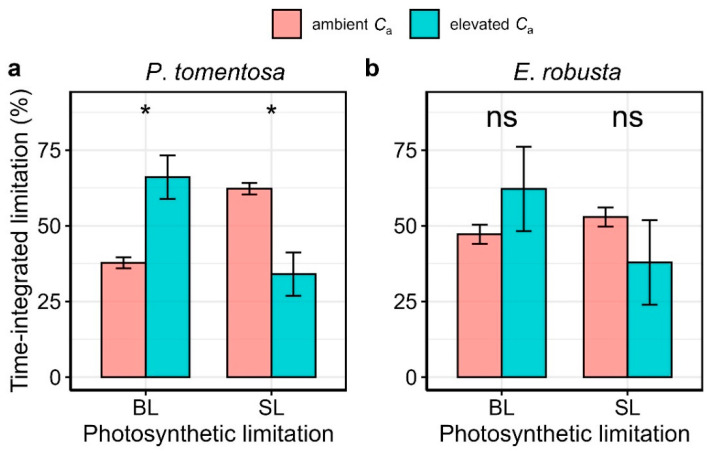
Quantitative time-integrated relative limitations by biochemical effects (BL) and stomatal effects (SL) under ambient and elevated *C*_a_ for *Populus tomentosa* (**a**) and *Eucalyptus robusta* (**b**). Asterisks (*) indicates significant differences in time-integrated relative limitations between ambient and elevated *C*_a_ at *p* < 0.05 according to the Wilcoxon signed-rank test, while ’ns’ denotes non-significant differences.

To further quantify the relative limitations of biochemical and stomatal effects during the photosynthetic induction, we partitioned the deviation of *A* from the final steady-state condition (d*A*_calc_) into biochemical limitation (d*A*_biochem_) and stomatal limitation (d*A*_stom_) over time, following Deans et al. (2019a, b) [10,30]. Given that elevated *C*_a_ may shift photosynthetic limitations to electron transport rate rather than being Rubico-limited, as originally assumed by Deans et al. (2019a, b) [10,30], we extended their framework to incorporate electron transport-limited conditions. As it is illustrated in Figure 5, biochemical and stomatal limitations were both initially strong, particularly in the early moments of induction, and gradually diminished as *A* approached steady-state values. Under ambient *C*_a_, time-integrated relative limitations revealed that biochemical and stomatal effects accounted for 62.25% and 37.75% of total photosynthetic limitations in *Populus tomentosa* and 52.85% and 47.15% in *E. robusta*, indicating a predominance of stomatal limitation in both species. In contrast, under elevated *C*_a_, stomatal limitations significantly (*p* < 0.05) decreased to 38.92% in *Populus tomentosa*, with a corresponding increase in biochemical limitation to 61.08% (Figure 6). Similar trends were observed in *E. robusta*, although changes were not statistically significant (*p* = 0.114), with relative stomatal limitation declining to 37.30% and biochemical limitation rising to 62.70%. These findings suggest that, under elevated CO_2_, biochemical limitations become the primary factor constraining photosynthetic induction in both species.

## 4. Discussion

### 4.1. Influence of Elevated CO_2_ on Relative Limitations of Biochemical and Stomatal Effects

Photosynthetic induction in plants is typically regulated by a balance between stomatal and biochemical limitations. Stomatal conductance (*g*_s_) has been widely studied as a limiting factor because its response to environmental changes is often an order of magnitude slower than photosynthesis [41,42]. Some studies identify *g*_s_ as the primary limitation to *A* [42,43], while others highlight biochemical limitation as dominant [32], with variation depending on species and the measurement conditions [44]. A recent analysis by Liu et al. (2022) [31] questioned the traditional approach of assessing limitation values at single time points, as Grassi et al. (2005) [3], and highlighted the application of a more dynamic, time-integrated approach to capture the progressive shifts in limitations [10,30]. In this study, we adopted the time-integrated framework of Deans et al. (2019a, b) [10,30] and extended it to account for the electron transport-limited photosynthesis under elevated CO_2_ (Figure 5 and Figure S1).

Consistent with prior studies, steady-state maximum carboxylation rate (*V*_cmax_) stomatal conductance (*g*_s_) declined under elevated CO_2_, likely contributing to photosynthetic acclimation and increase in intercellular CO_2_ concentration (*C*_i_), respectively [25,27,45,46,47]. During photosynthetic induction, our results demonstrate that elevated CO_2_ shifted the primary photosynthetic limitation from stomatal to biochemical processes in both *Populus tomentosa* and *Eucalyptus robusta* (Figure 6 and Figure S1). This shift aligns with findings according to which increased *C*_i_ due to greater stomatal opening can cause photosynthesis to become limited by electron transport [48]. Additionally, elevated CO_2_ reduced reliance on *g*_s_, enabling plants to sustain higher *C*_i_ levels with smaller stomatal openings [25,49]. We observed that, under elevated CO_2_, *g*_s_ initially increased to facilitate CO_2_ diffusion but subsequently stabilized at lower levels than under ambient *C*_a_, thereby reducing the stomatal limitations while maintaining higher intercellular CO_2_ concentration [4,45]. Notably, both ambient elevated *C*_a_ conditions shows such initial “overshooting” of stomatal opening, as described by McAuslan et al. (2016) [41] and Suwannarut et al. (2023) [50], though the overshoot was smaller under elevated CO_2_, suggesting reduced water loss and improved water use efficiency (WUE).

The reduction in stomatal limitation under elevated CO_2_ corresponded with an increased reliance on biochemical processes, particularly RuBP regeneration and electron transport, to meet the higher demand for carbon fixation. The increased availability of CO_2_ drives Rubisco closer to saturation, thus intensifying the demand for biochemical processes of RuBP regeneration to sustain enhanced photosynthetic rates [26,27,44]. This shift was evidenced by a reduction in time required to reach steady-state photosynthesis (*t*_90_) under elevated CO_2_ in both species, indicating faster photosynthetic induction when biochemical constraints dominate. This observation aligns with findings by Tomimatsu et al. (2016), who reported that biochemical limitations, especially electron transport and RuBP regeneration, become increasingly critical under elevated CO_2_ [34]. In our study, elevated CO_2_ increased *A* and significantly lowered steady-state *g*_s_, demonstrating enhanced photosynthetic efficiency through reduced stomatal constraints and prioritization of biochemical pathways.

Species-specific differences were evident, with *P. tomentosa* exhibiting more pronounced biochemical limitations under elevated CO_2_ than *E. robusta*. This may stem from inherent differences in baseline WUE and physiological responses. Known for its drought tolerance [51], *E. robusta* may have a less dynamic *g*_s_ response due to pre-adaptations for high WUE, resulting in more conservative *g*_s_ response to CO_2_ [25,52]. Such interspecies differences emphasize the importance of considering physiological variability when predicting ecosystem responses to rising atmospheric CO_2_.

### 4.2. Role of Photochemical and Non-Photochemical Quenching

Photochemical (PQ) and non-photochemical quenching (NPQ) play essential roles in managing light energy use and photoprotection, particularly under fluctuating light conditions. NPQ dissipates excess absorbed light energy as heat to prevent photodamage when light availability suddenly exceeds the capacity for photosynthetic utilization [53,54]. In our study, elevated CO_2_ was associated with a reduction in NPQ during the early moments of induction, suggesting less energy loss as heat and more energy was allocated to PQ, facilitating carbon fixation. This transition from photoprotection to productive photochemistry implies enhanced light-use efficiency under elevated CO_2_, reducing energy loss and enhancing photosynthetic efficiency [55,56].

Under elevated CO_2_, we observed a decrease in induction time (*t*_90_) for both the linear electron transport rate (*J*) and the fraction of open PSII centers (*q*_L_), potentially enabling more ATP and NADPH production to meet the biochemical demands of the Calvin cycle. Similar findings by Yamori et al. (2012) demonstrated that elevated CO_2_ allows plants to harness more light energy for carbon fixation, which is advantageous in environments with variable light [57]. By reallocating energy from NPQ to PQ, plants can improve photosynthetic performance, a trend that highlights the significant role PQ and NPQ dynamics play in determining photosynthetic limitations. The shift from stomatal to biochemical constraints under elevated CO_2_ appears to be supported by increased photochemical efficiency, underscoring the interdependence of light energy distribution and photosynthetic limitation processes [58,59].

Interestingly, species-specific differences in NPQ responses were also observed, with *P. tomentosa* exhibiting a more significant reduction in NPQ than *E. robusta* under elevated CO_2_. This differential response may reflect inherent differences in the species’ light-harvesting strategies and their ability to modulate photoprotection. *E. robusta* retained higher NPQ under fluctuating light, likely adopting a more conservative photoprotective approach to prevent photoinhibition in dynamic environments [60]. In contrast, *P. tomentosa* seems to maximize the benefits of elevated CO_2_ by reallocating energy from photoprotection to photochemistry, suggesting a species-specific adaptation to optimize carbon gain under high CO_2_ [25,61]. These findings highlight the importance of species-specific photochemical and non-photochemical responses in determining photosynthetic outcomes under elevated CO_2_.

### 4.3. Implications

Our findings reveal that elevated CO_2_ modulates the balance between stomatal and biochemical limitations during photosynthetic induction in both *Populus tomentosa* and *Eucalyptus robusta*, with implications for plant productivity and adaptability in fluctuating light environments. The shift from stomatal to biochemical limitations under elevated CO_2_ suggests that future atmospheric conditions may favor species with greater biochemical flexibility, enabling them to enhance carbon assimilation and minimize water loss, improving WUE [39,49,62,63]. This adaptation could prove particularly beneficial in arid and semi-arid ecosystems, where water availability is limited, allowing plants to achieve higher productivity with reduced reliance on stomatal regulation [27,33,58].

In addition, the reduced reliance on NPQ under elevated CO_2_ suggests that plants may become more resilient to photoinhibition in environments with variable light. By channeling more energy toward carbon fixation, plants in CO_2_-enriched atmospheres may maintain higher productivity and adapt more effectively to rapid light fluctuations [59,62]. This adaptability has important implications for agricultural productivity and ecosystem resilience, as enhanced photochemical efficiency and reduced photoprotective requirements may allow plants to exploit transient light events, such as sunflecks, which contribute significantly to overall carbon gain in dense canopies [9,57,64].

In summary, our study suggests a shift from stomatal to biochemical limitations under elevated CO_2_, highlighting a critical reorganization of physiological processes governing photosynthesis. This transition emphasizes the need to prioritize the role of biochemical flexibility and photochemical variability in understanding plant productivity under future atmospheric conditions. Considering such shifts in photosynthetic limitations is crucial for predicting plant response to rising CO_2_ and for developing strategies to optimize ecosystem productivity and resilience in a changing climate.

## Data Availability

The data that support the findings of this study are available from the corresponding author upon request.

## Supplementary Materials

The following supporting information can be downloaded at: https://www.mdpi.com/article/10.3390/plants14010047/s1, Figure S1. Biochemical and stomatal limitations to photosynthesis during photosynthetic induction under the assumption of Rubisco-limited (rather than electron transport rate limited) conditions at ambient and elevated *C*_a_ for *Populus tomentosa* and *Eucalyptus robusta*. Table S1. Mean values of steady-state parameters between ambient and elevated *C*_a_ for *Populus tomentosa* and *Eucalyptus robusta*.
