# Supplementary material for: Elevated CO2 Shifts Photosynthetic Constraint from Stomatal to Biochemical Limitations During Induction in Populus tomentosa and Eucalyptus robusta
Source: Plants (Basel). 2024 Dec 27;14(1):47. doi: 10.3390/plants14010047 (PMC11722825; doi:10.3390/plants14010047)
Supplement: Supplementary file 1 [file plants-14-00047-s001.zip › plants-3341260-supplementary.pdf]

Supplemental materials

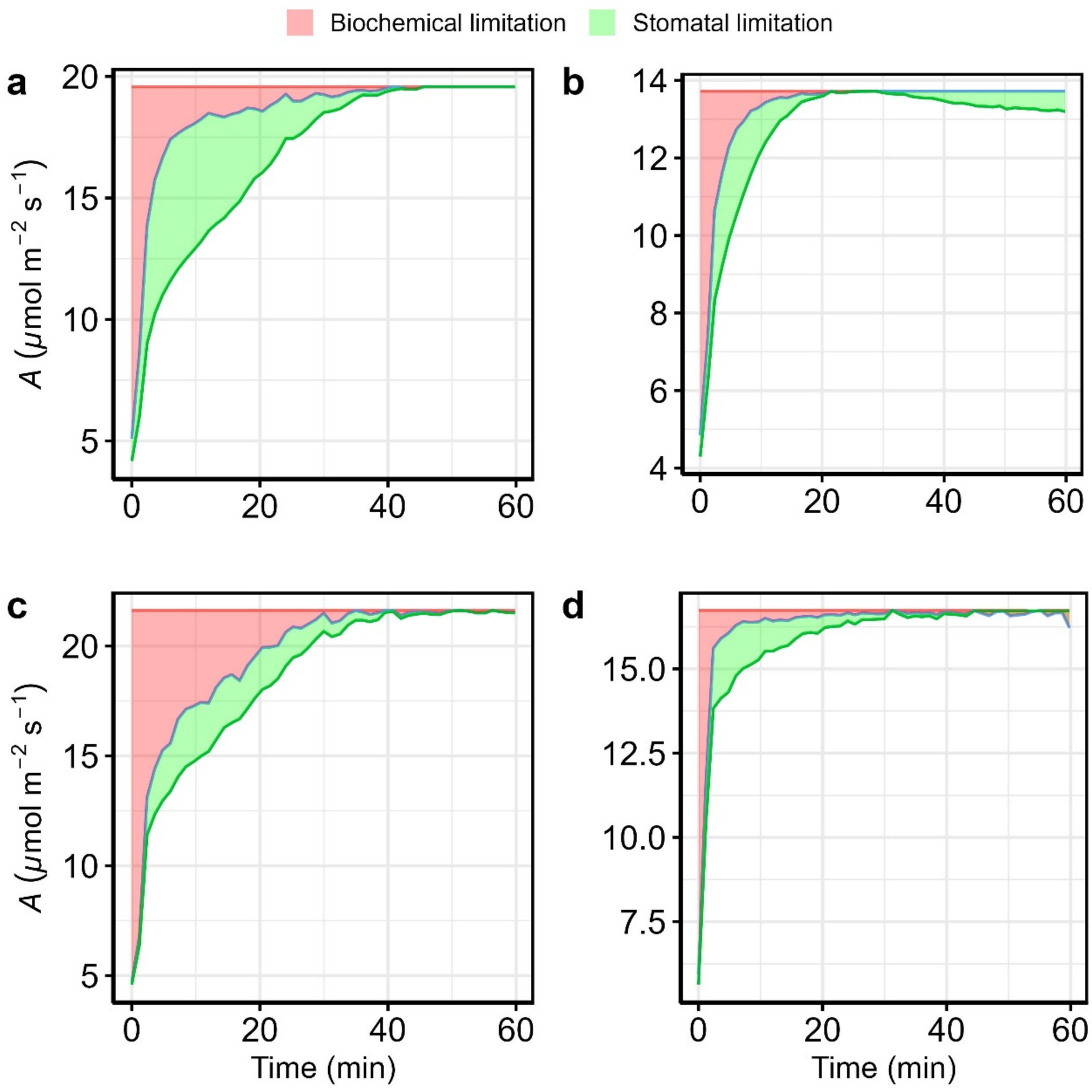

Figure S1 Biochemical and stomatal limitations to photosynthesis during photosynthetic induction under the assumption of Rubisco-limited (rather than electron transport rate limited) conditions at ambient C_a (upper panel) and elevated C_a (lower panel) for *Populus tomentosa* (left panel) and *Eucalyptus robusta* (right panel). At each time point, the deviation of A from the final steady-state (dA_{calc}) is divided into biochemical limitation ($dA_{biochem}$, red area, assuming Rubisco-limited photosynthesis) and stomatal limitation (dA_{stom}, green area). dA_{calc} represents the linearized difference between steady-state A and the current state A (dA), hence, $dA_{biochem}$ and dA_{stom} in the figures are normalized by multiplying dA/dA_{calc} for visual comparison.

Table S1 Mean values of steady state parameters between ambient and elevated C_a for *Populus tomentosa* and *Eucalyptus robusta*. Parameters marked with different letters are significantly different between ambient and elevated C_a at p<0.05.

Species	Treatments	Parameters in steady-state					
		A	g_s	V_{cmax}	J	q_L	NPQ
Populus tomentosa	ambient CO_2	19.29 ± 0.70 a	0.41 ± 0.05 a	67.16 ± 1.25 a	175.11 ± 2.58 a	0.42 ± 0.01 a	2.4 ± 0.03 a
	elevated CO_2	21.54 ± 1.21 a	0.17 ± 0.03 b	48.36 ± 1.48 b	166. 83 ± 4.07 a	0.42 ± 0.01 a	2.62 ± 0.02 b
Eucalyptus robusta	ambient CO_2	13.23 ± 0.76 a	0.23 ± 0.01 a	48.72 ± 2.98 a	88.19 ± 6.13 a	0.31 ± 0.02 a	2.9 ± 0.06 a
	elevated CO_2	16.64 ± 0.91 a	0.18 ± 0.02 b	35.78 ± 2.56 a	83.49 ± 5.78 a	0.29 ± 0.02 a	2.86 ± 0.09 a
